# Supplementary material for: Extracellular bacterial lymphatic metastasis drives Streptococcus pyogenes systemic infection
Source: Nat Commun. 2020 Sep 17;11:4697. doi: 10.1038/s41467-020-18454-0 (PMC7498588; doi:10.1038/s41467-020-18454-0)
Supplement: Supplementary file 1 — Supplementary Information [file 41467_2020_18454_MOESM1_ESM.pdf]

Supplementary Information

Supplementary Figures 1–7 and Supplementary Table 1

Extracellular bacterial lymphatic metastasis drives *Streptococcus pyogenes* systemic infection

Matthew K. Siggins[1,2]*, Nicola N. Lynskey[1,2], Lucy E. Lamb[1], Louise A. Johnson[3], Kristin K. Huse[1,2], Max Pearson[1,2], Suneale Banerji[3], Claire E. Turner[1], Kevin Woollard[4], David G. Jackson[3], Shiranee Sriskandan[1,2]*

*__Correspondence__: M.K.S (m.siggins@imperial.ac.uk) and S.S (s.sriskandan@imperial.ac.uk)

[1]Department of Infectious Disease, Imperial College London, W12 0NN, UK.

[2]MRC Centre for Molecular Bacteriology and Infection, Imperial College London, SW7 2DD, UK.

[3]MRC Human Immunology Unit, MRC Weatherall Institute of Molecular Medicine, University of Oxford, OX3 9DS, UK.

[4]Centre for Inflammatory Disease, Department of Immunology & Inflammation, Imperial College London, W12 0NN, UK.

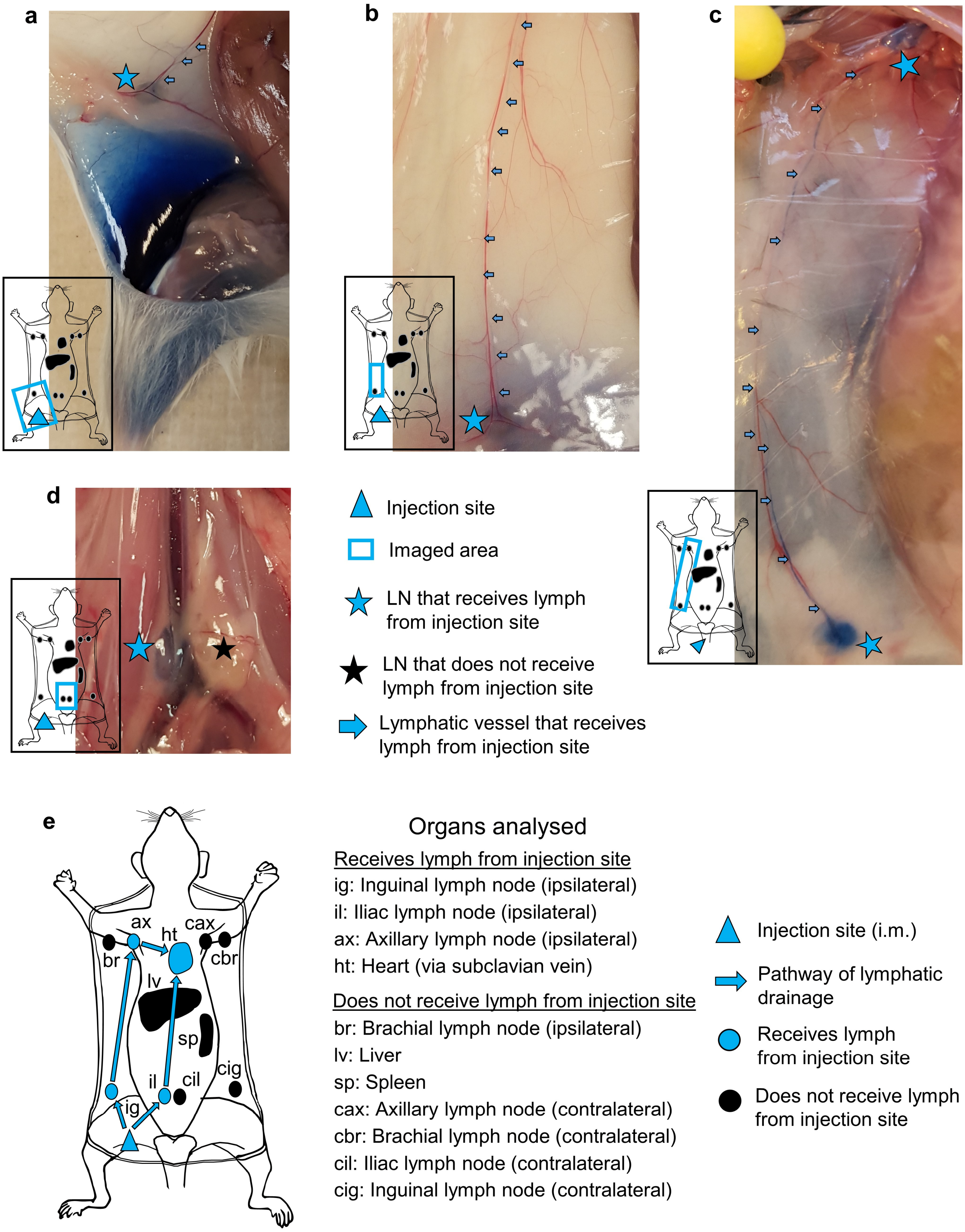

Supplementary Fig. 1 | Lymphatic drainage routes demonstrated by Evans Blue. a–**d**, Photographs showing drainage routes of Evans Blue dye following intramuscular injection into the hindleg (**a**, **b, d**) or subcutaneous injection into the tail (**c**) of mice. In-picture schematics show photographed area and injection site. **e**, Schematic showing summary of drainage following intramuscular injection into the hindleg. Data are representative of three independent experiments.

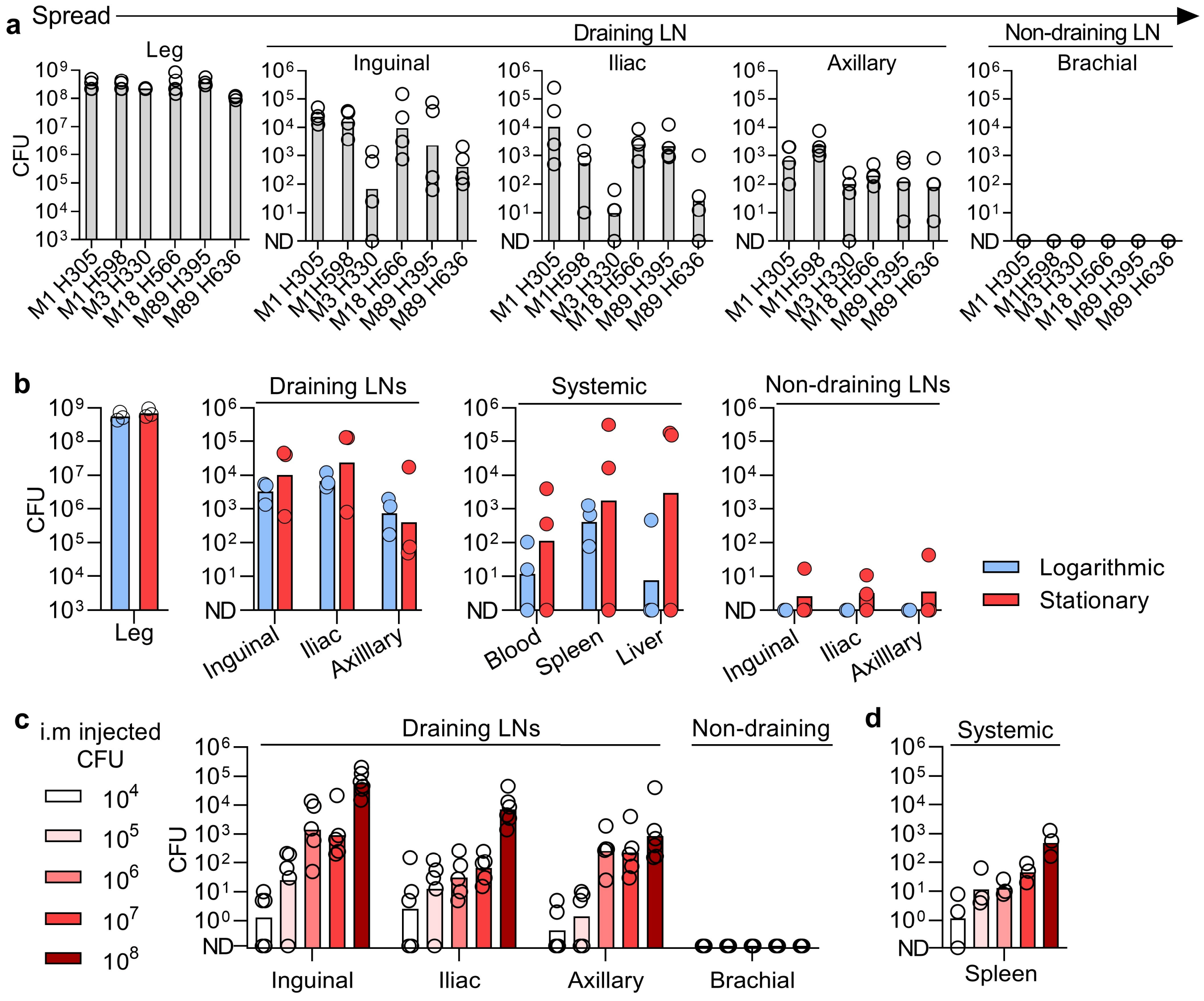

Supplementary Fig. 2 | Lymphatic-dominated dissemination of *S. pyogenes*. a, Bacterial counts recovered from the infection site, draining ipsilateral and non-draining ipsilateral lymph nodes of BALB/c mice, 3 h after intramuscular infection into the hindlimb with 10^8 CFU of a range of *S. pyogenes* isolates comprising of different *emm*-types. Symbols represent individual mice, *n* = 4 per group, and grey bar heights indicate geometric means. **b**, Bacterial counts recovered from the infection site, draining ipsilateral and non-draining ipsilateral lymph nodes of FVB/n mice, 3 h after intramuscular infection into the hindlimb with 10^8 CFU of logarithmic (blue) or stationary (red) phase *S. pyogenes* H598. Symbols represent individual mice, *n* = 3 per group, and bar heights indicate geometric means. **c**, **d** Bacterial counts recovered from draining- (inguinal, iliac, axillary) and non-draining (brachial) ipsilateral lymph nodes (**c**) and spleens (**d**) of FVB/n mice, 3 h after intramuscular infection into the hindlimb with 10^4–10^8 CFU, as stipulated, of hypervirulent *S. pyogenes* H598. Symbols represent individual mice, *n* = 5 or *n* = 3 (Spleen) per group and bar heights indicate geometric means. Source data are provided as a Source Data file.

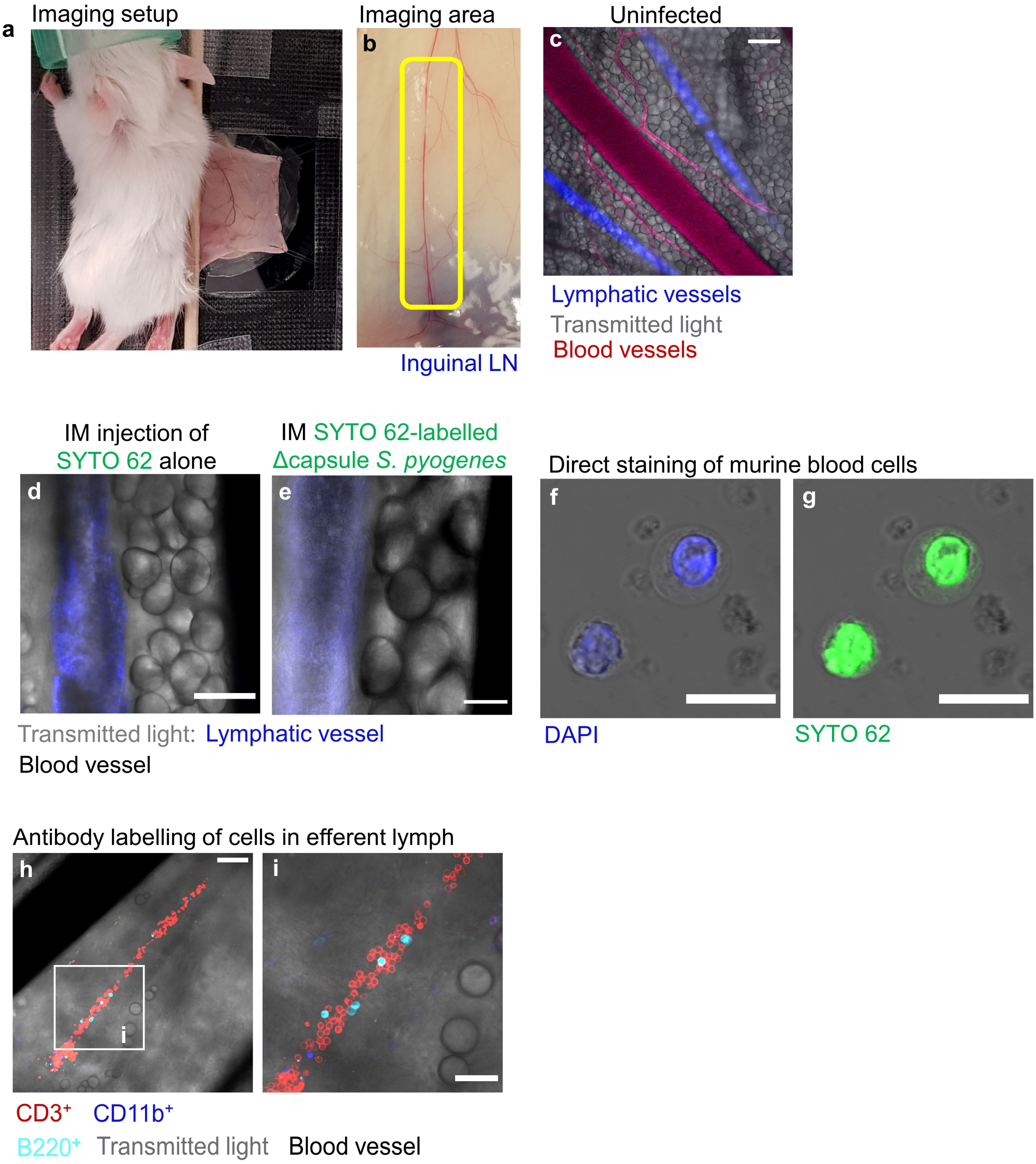

Supplementary Fig. 3 | Intravital confocal microscopy imaging of efferent lymphatic vessel. a, Photograph of anaesthetized mouse on microscope set up for imaging. **b**, Photograph of flank vasculature with imaging area, including the imaging area of the efferent lymphatic that links the inguinal and axillary lymph nodes, highlighted in yellow. **c**–**e**, Fluorescent and transmitted light intravital confocal microscopy image of an efferent lymphatic vessel between inguinal and axillary lymph nodes, prior to injection (**c**), or following intramuscular injection of 5 µM SYTO 62 (**d**) — a nucleic acid dye used to label bacteria in intravital microscopy experiments — or 10^8 CFU of SYTO 62 labelled capsule-deficient *S. pyogenes* (**e**). Lymph is labelled with FITC-conjugated dextran and appears blue (**c**–**e**), blood is labelled with TRITC-conjugated dextran and appears red (**c**). Scale bar

represents 200 µm. Supplementary Movie 1 shows blood and lymph flow in distinct vessels in the flank. **f**, **g**, Murine blood cells labelled directly with 30 µM DAPI (**f**), or 5 µM SYTO 62 (**g**), both demonstrate concentrated staining of nucleus without cytoplasmic or punctate staining. Scale bars represent 10 µm. **h**, **i**, Representative fluorescent and transmitted light intravital confocal microscopy image of an efferent lymphatic vessel and blood vessels with cells stained for CD3 (red), B220 (cyan), and CD11b (blue) after infection with hypervirulent *S. pyogenes*. Scale bar represents 200 µm (**h**) and 100 µm (**i**). Imaging data are representative of three independent experiments.

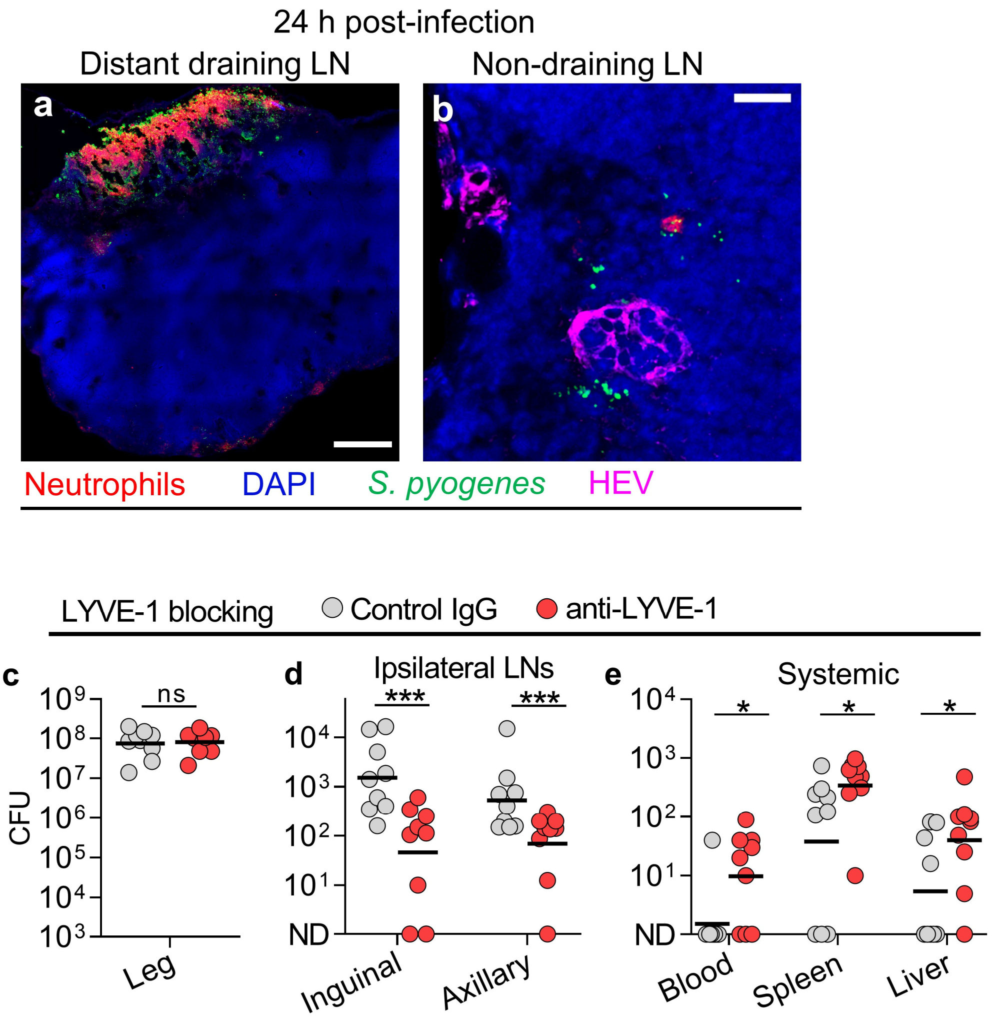

Supplementary Fig. 4 | Dissemination of *S. pyogenes* in late infection. a, **b**, Immunofluorescence staining of cryosections from the distant draining (**a**) and non-draining (**b**) axillary lymph nodes of FVB/n mice 24 h after intramuscular infection into the hindlimb with 10^8 CFU of *S. pyogenes* H1565 (green); Neutrophils (red); DAPI (blue); and High Endothelial Venules (magenta, **c** only). Scale bars: 200 µm (**a**), 50 µm (**b**). Imaging data are representative of five independent experiments. **LYVE-1 augments retention of encapsulated *S. pyogenes* within lymph nodes. c**–**e,** Blocking LYVE-1 impairs lymphatic-retention of encapsulated *S. pyogenes* and promotes systemic bacterial spread: High-Capsule ΔP2 (H1458) recovered from the infection site (**c**), ipsilateral draining-lymph nodes (**d**), or systemic organs (**e**) of FVB/n mice injected intraperitoneally with a LYVE-1 blocking (red circles) or control antibody (grey circles) 24 h prior to a 3-h intramuscular infection with 10^8 CFU. Symbols represent individual mice, $n = 9$ per group, black lines indicate geometric means. *$p \leq 0.05$; ***$p \leq 0.001$; ns, $p > 0.5$. Inguinal, $p = 0.0009$; Axillary, $p = 0.0070$; Blood, $p = 0.0430$; Spleen, $p = 0.0140$; Liver, $p = 0.0243$; Two-tailed Mann-Whitney test. Source data are provided as a Source Data file.

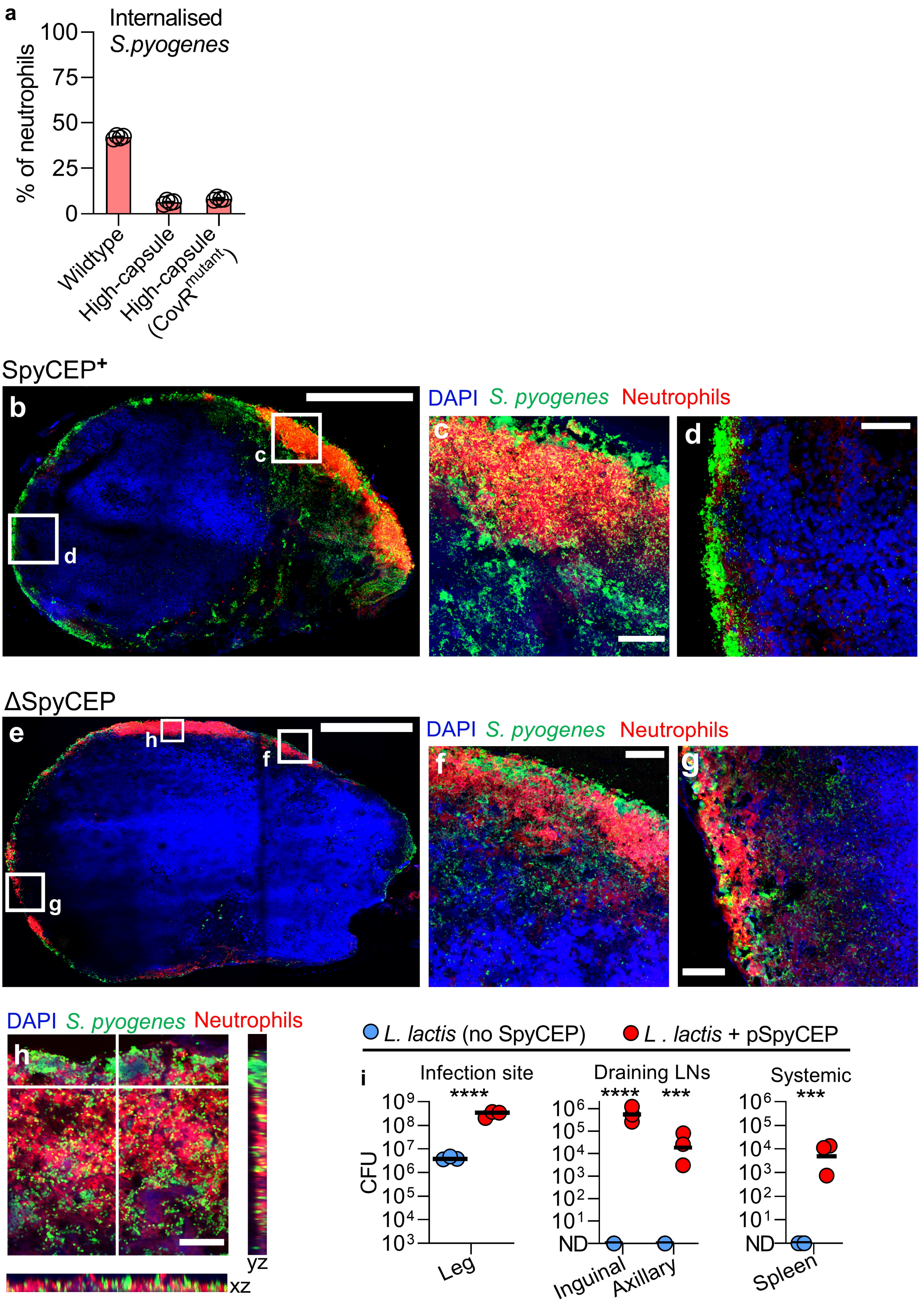

Supplementary Fig. 5 | Role of SpyCEP and neutrophils in lymphatic dissemination. a, Internalisation of *S. pyogenes* strains by human neutrophils after 30 minutes co-incubation at a multiplicity of infection of 10 bacteria: 1 neutrophil, measured by flow cytometry. Symbols represent

individual data points, *n* = 4; height of red bars indicate means and error bars represent standard error of the mean. **b**–**g,** Immunofluorescence images of cryosections from the local draining lymph nodes of FVB/n mice 24 h after intramuscular infection into the hindlimb with 10^8 CFU of *S. pyogenes* $SpyCEP^+$ H1565 (**b**–**d)** or ΔSpyCEP H1567 (**e**–**g**). Orthogonal views of a 12 µm confocal z-stack (**h**); *S. pyogenes* (green), neutrophils (red), DAPI (blue). Scale bars: 20 µm (**h**), 50 µm (**c**, **d**, **f**, **g**), 500 µm (**b**, **e**). Imaging data are representative of five independent experiments. **i,** Bacterial counts recovered from the infection site, draining lymph nodes and spleens of C57BL/6 mice, 3 h after intramuscular infection into the hindlimb with 10^8 CFU of wildtype *L. lactis* (blue circles), or *L. lactis* expressing SpyCEP (red circles). Symbols represent individual mice, *n* = 3 per group, and black lines indicate geometric means. ****p ≤ 0.0001 ***p ≤ 0.001. Leg, p = <0.0001; Inguinal, p = <0.0001; Axillary, p = 0.0005; Spleen, p = 0.0008; Two-tailed Student's t-test performed on $\log_{10}$-transformed data. Source data are provided as a Source Data file.

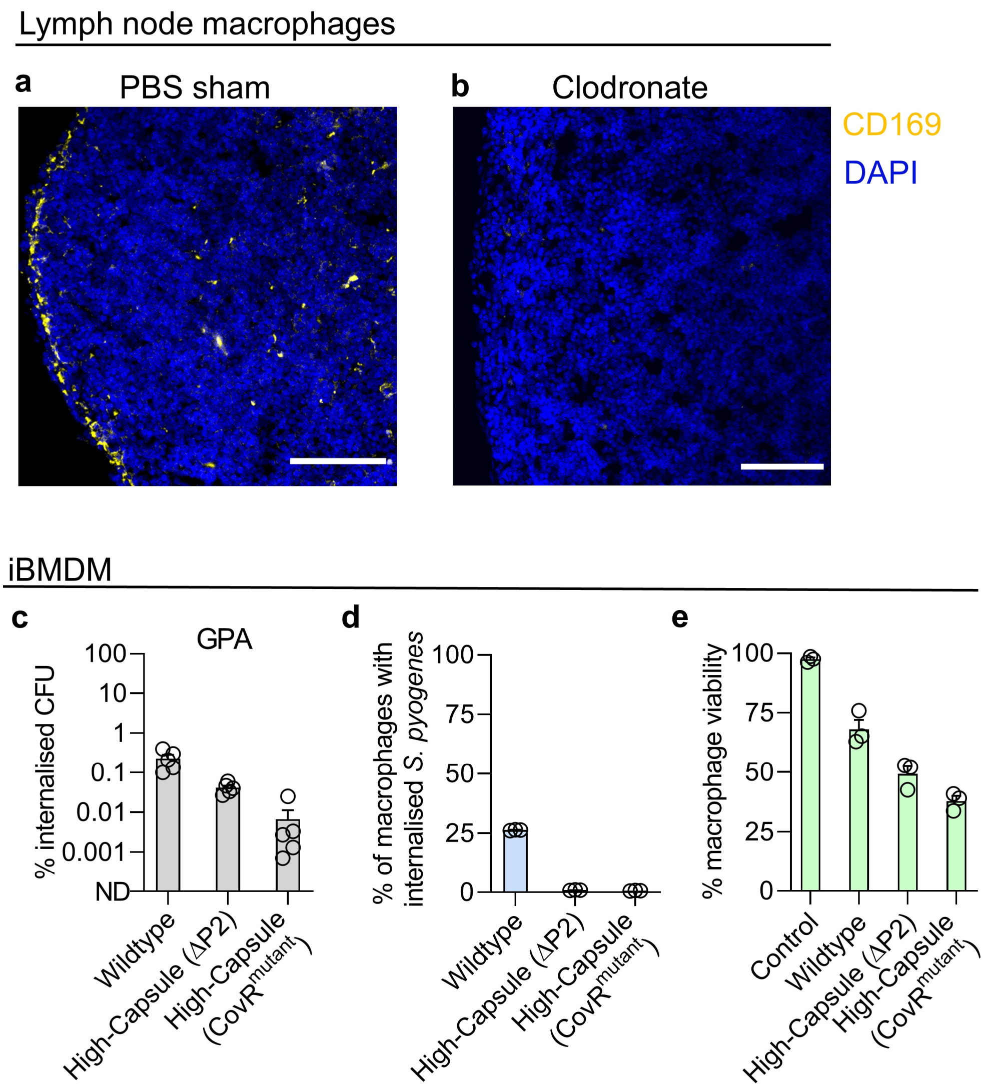

Supplementary Fig. 6 | Macrophages exhibit limited phagocytosis and killing of *S. pyogenes*. a, **b**, Immunofluorescence staining of CD169^{+} macrophages in cryosections from the local draining lymph nodes of FVB/n mice 96 h after hindlimb and tail subcutaneous injections of PBS (**a**) or clodronate liposomes (**b**); CD169 (yellow), DAPI (blue) Scale bars: 100 µm. Imaging data are representative of three independent experiments **c**, grey bars show viable intracellular *S. pyogenes* strains as a percentage of original inoculum, determined by gentamicin protection assay (GPA) following 3 h of co-incubation of *S. pyogenes* with murine immortalised bone marrow-derived macrophages (multiplicity of infection 10:1). **d, e,** percentage of macrophages with internalised *S. pyogenes* (blue bars, **d**) and macrophage viability (**e**, green bars), both measured by flow cytometry after 3 h of co-incubation of *S. pyogenes* with murine immortalised bone marrow-derived macrophages (multiplicity of infection 10:1). Symbols represent individual data points, n = 5 (**a**) or n = 3 (**b**, **c**); bar height indicates mean and error bars represent standard error of the mean. Source data are provided as a Source Data file.

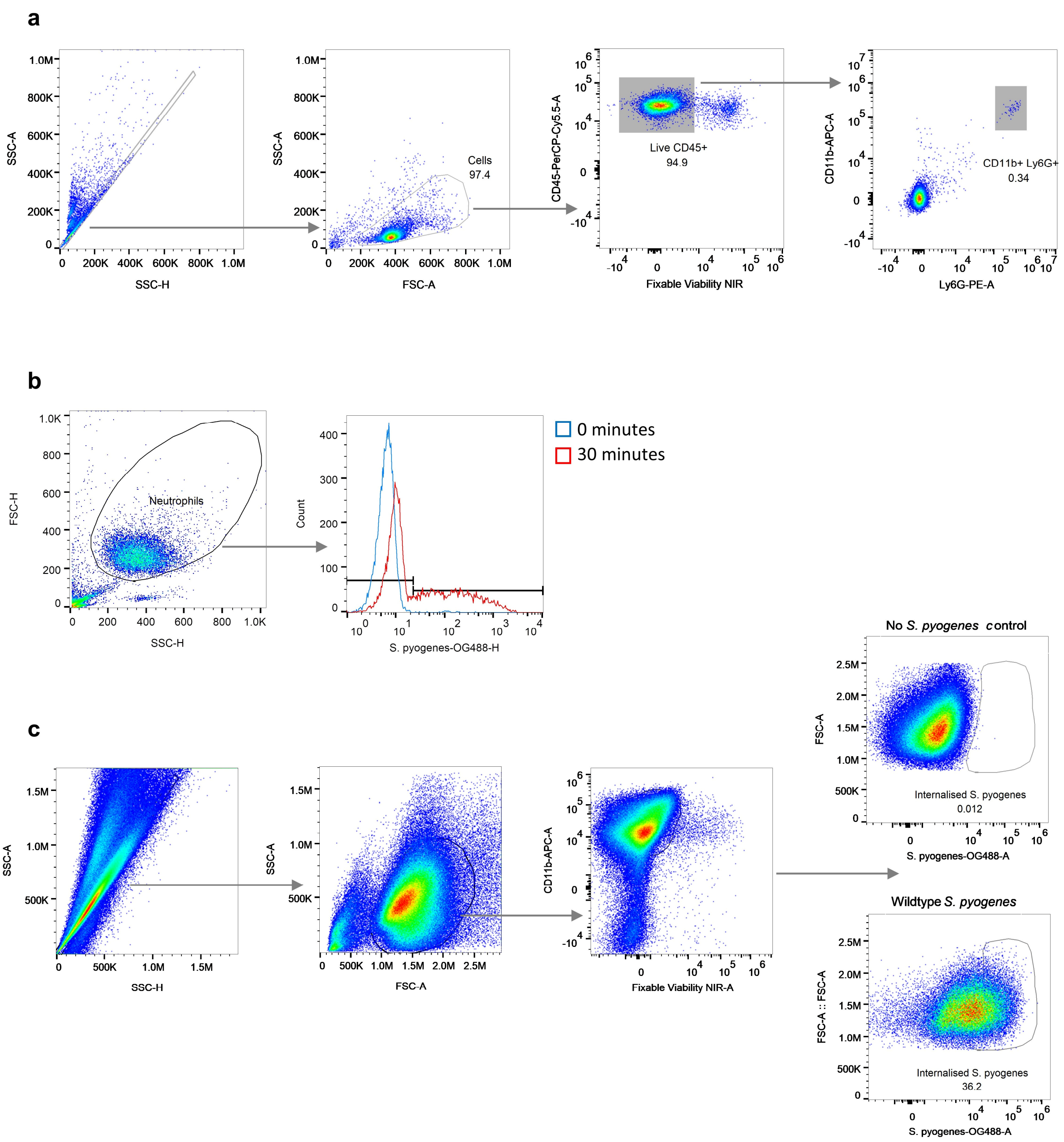

Supplementary Fig. 7 | Representative gating strategies for flow cytometry. a, single cell suspensions from lymph nodes and blood were gated by singlets, size, then live CD45$^+$ cells were determined. Neutrophils were identified by further gating on CD11b and Ly6G: approach used for Fig. 5b. **b**, Neutrophils isolated from human blood were gated by size and then levels of internalised fluorescently-labelled *S. pyogenes* measured by gating on OG-488: approach used for Supplementary Fig. 5a. **c**, cultured iBMDM were gated by singlets, size, then live CD11b$^+$ cells were determined. Level of internalised fluorescently labelled *S. pyogenes* was measured in these cells by gating on OG-488: approach used for Supplementary Fig. 6d, e.

Supplementary Table 1 | Bacterial strains used in this study

**Species**	**Strain ID**	***emm* type / strain**	**Description**	**Reference**
S. pyogenes	H598	*emm*1	Hypervirulent, M1T1 invasive necrotising fasciitis isolate with natural *covS* mutation	[1]
S. pyogenes	H305	*emm*1	Scarlet fever M1T1 throat isolate (NCTC8198)	[2]
S. pyogenes	H330	*emm*3	Invasive puerperal sepsis blood isolate	[3]
S. pyogenes	H395	*emm*89	Invasive pneumonia blood isolate	[4]
S. pyogenes	H566	*emm*18	Invasive isolate, naturally highly encapsulated	[5]
S. pyogenes	H636	*emm*89	Invasive chest sepsis isolate	[4]
S. pyogenes	H584	*emm*1	M1T1 invasive puerperal sepsis blood isolate	[1]
S. pyogenes	H1454	*emm*1	Hyaluronan capsule deletion mutant of H584	This study
S. pyogenes	H1458	*emm*1	Highly encapsulated, hyaluronan capsule promoter P2 mutant of H584, selected in lymph node in vivo	[6]
S. pyogenes	H1565	*emm*1	Hypervirulent, highly encapsulated mutant of H584 with *covR* mutation, selected in spleen in vivo	[6]
S. pyogenes	H1567	*emm*1	SpyCEP deletion mutant of H1565	This study
E. coli		XL-10 gold		Agilent
L. lactis	H486	NZ9000	with control plasmid pDestErm	[7]
L. lactis	H487	NZ9000	with SpyCEP expression plasmid p*cepA*	[7]

Blue shaded cells indicate *S. pyogenes* H584 parent and derived strains.

Supplementary References

1. Turner CE*, et al.* Molecular analysis of an outbreak of lethal postpartum sepsis caused by Streptococcus pyogenes. *J Clin Microbiol* **51**, 2089-2095 (2013).

2. Sriskandan S, Unnikrishnan M, Krausz T, Cohen J. Molecular analysis of the role of streptococcal pyrogenic Exotoxin A (SPEA) in invasive soft-tissue infection resulting from Streptococcus pyogenes. *Mol Microbiol* **33**, 778-790 (1999).

3. Afshar B*, et al.* Enhanced nasopharyngeal infection and shedding associated with an epidemic lineage of emm3 group A Streptococcus. *Virulence*, 1-11 (2017).

4. Turner CE*, et al.* Emergence of a New Highly Successful Acapsular Group A Streptococcus Clade of Genotype emm89 in the United Kingdom. *MBio* **6**, e00622 (2015).

5. Lynskey NN*, et al.* RocA truncation underpins hyper-encapsulation, carriage longevity and transmissibility of serotype M18 group A streptococci. *PLoS Pathog* **9**, e1003842 (2013).

6. Lamb LE*, et al.* Impact of contusion injury on intramuscular emm1 group a streptococcus infection and lymphatic spread. *Virulence* **9**, 1074-1084 (2018).

7. Zinkernagel AS*, et al.* The IL-8 protease SpyCEP/ScpC of group A Streptococcus promotes resistance to neutrophil killing. *Cell Host Microbe* **4**, 170-178 (2008).
