# Supplementary material for: Extracellular bacterial lymphatic metastasis drives Streptococcus pyogenes systemic infection
Source: Nat Commun. 2020 Sep 17;11:4697. doi: 10.1038/s41467-020-18454-0 (PMC7498588; doi:10.1038/s41467-020-18454-0)
Supplement: Supplementary file 3 — Description of Additional Supplementary Files [file 41467_2020_18454_MOESM3_ESM.docx]

**Description of Additional Supplementary Files**

Extracellular bacterial lymphatic metastasis drives *Streptococcus pyogenes* systemic infection

Supplementary Movies 1–9: Descriptions and Legends

**File Name**: Supplementary Movie 1

**Description**: Lymph flow in efferent lymphatic vessel.

Representative fluorescent and transmitted light intravital confocal microscopy time series video of lymph, labelled with FITC-conjugated dextran (blue), and cells (dark grey) flowing in the efferent lymphatic vessel that connects the inguinal and axillary lymph nodes. Blood flow, labelled with TRITC-conjugated dextran (red), also maintained. Scale bar represents 75 µm.

**File Name**: Supplementary Movie 2

**Description**: *S. pyogenes* transiting in efferent lymph.

Representative fluorescent intravital confocal microscopy time series video of *S. pyogenes* transiting (circled) in the efferent lymphatic between the inguinal and axillary lymph nodes. Mouse cells labelled with Hoechst 33342 and appear red; Lymph labelled with FITC-conjugated dextran appears blue; and fluorescent *S. pyogenes*, injected intramuscularly into the hind leg prior to imaging, appear green. Scale bar 20 µm.

**File Name**: Supplementary Movie 3

**Description**: Cells with surface-adhered *S. pyogenes* transiting in efferent lymph flow.

Representative fluorescent intravital confocal microscopy time series video of cells with adherent *S. pyogenes* transiting in the efferent lymphatic between the inguinal and axillary lymph nodes. Some streptococci appear adhered to the vessel walls. Lymph labelled with FITC-conjugated dextran appears blue, and fluorescent *S. pyogenes*, injected intramuscularly into the hind leg prior to imaging, appear green. Scale bar 20 µm.

**File Name**: Supplementary Movie 4

Description: *S. pyogenes* within efferent lymphatics are extracellular.

Three-dimensional reconstruction of images from fluorescent and transmitted light intravital confocal microscopy z-stack series, shows *S. pyogenes* are extracellular within an efferent lymphatic vessel. Lymph labelled with FITC-conjugated dextran appears blue, and fluorescent *S. pyogenes*, injected intramuscularly into the hind leg prior to imaging, appear green. Scale bar represents 10 µm.

**File Name**: Supplementary Movie 5

Description: Characteristic *S. pyogenes* chains visible on leukocyte cell surface within efferent lymphatics.

Magnified section of Supplementary Movie 4 which shows a three-dimensional reconstruction of chains of *S. pyogenes* associated with the cell surface of a leukocyte within an efferent lymphatic. Lymph labelled with FITC-conjugated dextran appears blue, and fluorescent *S. pyogenes*, injected intramuscularly into the hind leg prior to imaging, appear green. Scale bar represents 10 µm.

**File Name**: Supplementary Movie 6

Description: *S. pyogenes* accumulates in peripheral sinuses of local-draining inguinal lymph node.

Immunofluorescence confocal microscopy pan and zoom video of a maximum projection image, generated from a z-stack of a 10 µm cryosection, of the local-draining ipsilateral inguinal lymph node 3 h after hind leg intramuscular infection with hypervirulent *S. pyogenes* (H1565). Bacteria (green) accumulate in podoplanin (magenta) -positive subcapsular and medullary sinuses at the periphery of the lymph node, but not in interior cortical areas, indicating nodal entry and exit occurs through lymphatic vessels. Podoplanin staining in the interior of the node is due to the presence of fibroblastic reticular cells. Cell nuclei stained with DAPI (blue).

**File Name**: Supplementary Movie 7

Description: *S. pyogenes* accumulates in peripheral sinuses of distant-draining axillary lymph node.

Immunofluorescence confocal microscopy pan and zoom video of a maximum projection image, generated from a confocal microscopy z-stack of a 10 µm cryosection, of the distant-draining ipsilateral axillary lymph node 3 h after hind leg intramuscular infection with hypervirulent *S. pyogenes* (H1565). Bacteria (green) accumulate in podoplanin (magenta) -positive subcapsular and medullary sinuses at the periphery of the lymph node, but not in interior cortical areas, indicating nodal entry and exit occurs through lymphatic vessels. Podoplanin staining in the interior of the node is due to the presence of fibroblastic reticular cells. Cell nuclei stained with DAPI (blue).

**File Name**: Supplementary Movie 8

Description: Hyper-virulent *S. pyogenes* restrict neutrophil recruitment in draining lymph nodes.

Immunofluorescence pan and zoom video of a maximum projection image, generated using confocal microscopy through a 10 µm cryosection, of the local-draining ipsilateral inguinal lymph node 24 h after hind leg intramuscular infection with hypervirulent *S. pyogenes* (H1565). Many neutrophils (magenta) surround *S. pyogenes* (green) in most peripheral sinuses. However, in deeper medullary sinuses there are few neutrophils in relation to the number of bacteria. In addition, a large area of high bacterial density shows relatively few neutrophils and diffuse DAPI (blue) staining, indicating significant host cell damage. Scale bar represents 10 µm.

**File Name**: Supplementary Movie 9

Description: *S. pyogenes* are minimally sequestered by phagocytic cells within lymph nodes.

Immunofluorescence confocal microscopy z-stack series, generated from a 10 µm cryosection, of the local-draining ipsilateral inguinal lymph node 3 h after hind leg intramuscular infection with hypervirulent *S. pyogenes* (H1565). Streptococci (green) are extremely infrequently colocalized with or inside CD169^+^ macrophages (blue). Some phagocytosis by neutrophils (Ly6G^+^, red) is present, but largely bacteria are not associated with these cells either. Scale bar represents 10 µm.
