# Supplementary material for: Extracellular bacterial lymphatic metastasis drives Streptococcus pyogenes systemic infection
Source: Nat Commun. 2020 Sep 17;11:4697. doi: 10.1038/s41467-020-18454-0 (PMC7498588; doi:10.1038/s41467-020-18454-0)
Supplement: Supplementary file 13 — Reporting Summary [file 41467_2020_18454_MOESM13_ESM.pdf]

nature research

Corresponding author(s): Dr Matthew Siggins
Professor Shiranee Sriskandan

Last updated by author(s): Aug 14, 2020

# Reporting Summary

Nature Research wishes to improve the reproducibility of the work that we publish. This form provides structure for consistency and transparency in reporting. For further information on Nature Research policies, see our Editorial Policies and the Editorial Policy Checklist.

## Statistics

For all statistical analyses, confirm that the following items are present in the figure legend, table legend, main text, or Methods section.

| n/a | Confirmed | |
|---|---|---|
| [ ] | [x] | The exact sample size ($n$) for each experimental group/condition, given as a discrete number and unit of measurement |
| [ ] | [x] | A statement on whether measurements were taken from distinct samples or whether the same sample was measured repeatedly |
| [ ] | [x] | The statistical test(s) used AND whether they are one- or two-sided<br>*Only common tests should be described solely by name; describe more complex techniques in the Methods section.* |
| [x] | [ ] | A description of all covariates tested |
| [ ] | [x] | A description of any assumptions or corrections, such as tests of normality and adjustment for multiple comparisons |
| [ ] | [x] | A full description of the statistical parameters including central tendency (e.g. means) or other basic estimates (e.g. regression coefficient) AND variation (e.g. standard deviation) or associated estimates of uncertainty (e.g. confidence intervals) |
| [ ] | [x] | For null hypothesis testing, the test statistic (e.g. $F$, $t$, $r$) with confidence intervals, effect sizes, degrees of freedom and $P$ value noted<br>*Give P values as exact values whenever suitable.* |
| [x] | [ ] | For Bayesian analysis, information on the choice of priors and Markov chain Monte Carlo settings |
| [x] | [ ] | For hierarchical and complex designs, identification of the appropriate level for tests and full reporting of outcomes |
| [x] | [ ] | Estimates of effect sizes (e.g. Cohen's $d$, Pearson's $r$), indicating how they were calculated |

*Our web collection on statistics for biologists contains articles on many of the points above.*

## Software and code

Policy information about availability of computer code

| | |
|---|---|
| Data collection | Leica LAS – Version 4: leica-microsystems.com/products/microscope-software/p/leica-application-suite<br>CytExpert – Version 2.4: mybeckman.uk/flow-cytometry/instruments/cytoflex/software<br>BD CellQuest Pro – Version 5.1<br>Attune NxT Software – Version 4.2: thermofisher.com/uk/en/home/global/forms/attune-nxt-software-download-registration.html |
| Data analysis | Graphpad Prism – Version 8.4: graphpad.com/scientific-software/prism/<br>ImageJ-Fiji – Version 2.0.0-rc-68/1.52v: imagej.net/Fiji/Downloads<br>FlowJo – Version 10.6.2: flowjo.com/solutions/flowjo/downloads<br>Leica Application Suite X – Version 3.7.0: leica-microsystems.com/products/microscope-software/p/leica-las-x-ls/<br>GIMP 2.10.18: gimp.org/downloads |

For manuscripts utilizing custom algorithms or software that are central to the research but not yet described in published literature, software must be made available to editors and reviewers. We strongly encourage code deposition in a community repository (e.g. GitHub). See the Nature Research guidelines for submitting code & software for further information.

## Data

Policy information about availability of data

All manuscripts must include a data availability statement. This statement should provide the following information, where applicable:

- Accession codes, unique identifiers, or web links for publicly available datasets
- A list of figures that have associated raw data
- A description of any restrictions on data availability

Source data are provided with this paper. Further data that support the findings of this study are available from the corresponding authors upon reasonable request

# Field-specific reporting

Please select the one below that is the best fit for your research. If you are not sure, read the appropriate sections before making your selection.

[x] Life sciences [ ] Behavioural & social sciences [ ] Ecological, evolutionary & environmental sciences

For a reference copy of the document with all sections, see nature.com/documents/nr-reporting-summary-flat.pdf

# Life sciences study design

All studies must disclose on these points even when the disclosure is negative.

| | |
|---|---|
| Sample size | G*Power 3.1.9.7 was used to calculate group size using Alpha error = 0.05 and Power = 0.8 for and effect sizes determined from previous data and pilot experiments. |
| Data exclusions | No data were excluded |
| Replication | At least experimental triplicates were included, with most experiments being performed more than once with concordant results. All attempts of replication, including by other researchers have been successful. |
| Randomization | For all mouse experiments the distribution of age and gender was matched between experimental groups. For experiments with human cells or blood, all experimental groups used cells from the same donors. |
| Blinding | Blinding using two operators and/or coded samples was performed for immunofluorescence and intravital experiments. However, for other experiments this was not possible as the sole investigator performing the experiments was also required to conduct analysis. |

# Reporting for specific materials, systems and methods

We require information from authors about some types of materials, experimental systems and methods used in many studies. Here, indicate whether each material, system or method listed is relevant to your study. If you are not sure if a list item applies to your research, read the appropriate section before selecting a response.

### Materials & experimental systems

| n/a | Involved in the study | |
|---|---|---|
| [ ] | [x] | Antibodies |
| [ ] | [x] | Eukaryotic cell lines |
| [x] | [ ] | Palaeontology and archaeology |
| [ ] | [x] | Animals and other organisms |
| [ ] | [x] | Human research participants |
| [x] | [ ] | Clinical data |
| [x] | [ ] | Dual use research of concern |

### Methods

| n/a | Involved in the study | |
|---|---|---|
| [x] | [ ] | ChIP-seq |
| [ ] | [x] | Flow cytometry |
| [x] | [ ] | MRI-based neuroimaging |

## Antibodies

Antibodies used

IF: anti-Ly6G-AF594 (1A8, BioLegend #127636); anti-CD169-AF647 (3D6.112, BioLegend #142408); anti-PNAd-biotin (MECA-79, BioLegend #120804); hamster anti-podoplanin (8.1.1, eBioscience #14-5381-82); anti-LYVE-1-eFluor 570 (ALY7, Invitrogen, #41-0443-822), anti-Group A Carbohydrate-FITC (abcam #ab9191), anti-hamster IgG-AF647 (Invitrogen #A-21451)and Streptavidin-AF546 (Invitrogen #S11225).

IVM: anti-B220 Brilliant Violet 421 (RA3-6B2, BioLegend #103251), anti-CD3-PE (145-2C11, BioLegend #100308), anti-CD11b-Alexa Fluor 488 (M1/70, BioLegend #101217), anti-F4/80- Brilliant Violet 421 (BM8, BioLegend #123137).

FC: anti-CD16/32 (93, BioLegend #101302), anti-CD45-PerCP-Cy5.5 (30-F11, BioLegend #103132), anti-CD11b-APC (M1/70, BioLegend #101212), anti-Ly6-G-PE (1A8, BioLegend #127608), anti-CD11c-FITC (N418, Biolegend #117306), anti-CD169-PE (REA197,

| | |
|---|---|
| | Miltenyi Biotec #130-124-896) and anti-F4/80-FITC (REA126, Miltenyi Biotec #130-117-509). |
| Validation | Antibodies were purchased from commercial manufacturers, who validated performance for antigen and species specificity for flow cytometry and immunofluorescence staining. All validation statements, citations, quality control, and further details are found on manufacturer websites. |

## Eukaryotic cell lines

Policy information about cell lines

| | |
|---|---|
| Cell line source(s) | Immortalised bone marrow-derived macrophages were obtained from Dr Paras Anand |
| Authentication | Characteristics of immortalised bone marrow-derived macrophages were confirmed by flow cytometry |
| Mycoplasma contamination | Cells were not mycoplasma positive |
| Commonly misidentified lines (See ICLAC register) | No commonly misidentified cell lines were used in this study. |

## Animals and other organisms

Policy information about studies involving animals; ARRIVE guidelines recommended for reporting animal research

| | |
|---|---|
| Laboratory animals | 4–10-week old FVB/n, BALB/c and C57BL/6 female mice acquired from Charles River UK were used. Mice were housed in standard conditions, ambient temperature of 21ºC, 50% humidity and a 12-hour light cycle. |
| Wild animals | The study did not involve wild animals |
| Field-collected samples | The study did not involve field-collected samples |
| Ethics oversight | In vivo experiments were performed in accordance with the Animal (Scientific Procedures) Act 1986, with appropriate UK Home Office licenses according to established institutional guidelines |

Note that full information on the approval of the study protocol must also be provided in the manuscript.

## Human research participants

Policy information about studies involving human research participants

| | |
|---|---|
| Population characteristics | Fresh human neutrophils were acquired from an normal healthy donors consisting of males and females, ages 18–38 |
| Recruitment | Donors were healthy volunteers recruited from employees and students of Imperial College London. All experimental groups within an experiment used cells from the same blood donor and so any potential selection bias would not impact results. |
| Ethics oversight | Approved subcollection of normal healthy donor blood samples of the Imperial College London NIHR Biomedical Research Centre Tissue Bank, all volunteers gave informed consent |

Note that full information on the approval of the study protocol must also be provided in the manuscript.

## Flow Cytometry

### Plots

Confirm that:

- [x] The axis labels state the marker and fluorochrome used (e.g. CD4-FITC).
- [x] The axis scales are clearly visible. Include numbers along axes only for bottom left plot of group (a 'group' is an analysis of identical markers).
- [x] All plots are contour plots with outliers or pseudocolor plots.
- [x] A numerical value for number of cells or percentage (with statistics) is provided.

### Methodology

| | |
|---|---|
| Sample preparation | Neutrophils were isolated from heparinised human blood using a MACSXpress Whole Blood Neutrophil Isolation Kit (Miltenyi Biotec) and resuspened in HBSS. Dissected lymph nodes were disrupted with a motorised pellet pestle and then chopped further with scissors. Dissected spleens were minced with scissors. Lymph node and spleen fragments were then digested for 20 minutes at 37°C with 200 U/ml collagenase IV and 100 U/ml DNase I (both Worthington Biochemical Corp) in digestion buffer (HBSS with 0.5% BSA, 2 mM Ca2+ and 10 mM HEPES). Spleens were next passed through a 100 µm cell strainer in cell buffer (HBSS with 2 mM EDTA, 0.5% BSA, and 10 mM HEPES) containing 100 U/ml DNase I. For lymph nodes, large tissue fragments were allowed to settle and the supernatant, which contained the cells of interest, was removed and the cells washed before resuspension in cell buffer. If required, erythrocytes in spleen suspensions and blood were lysed with BD Pharm Lyse (BD Biosciences). Single cell suspensions from the organs and blood of mice were resuspended in PBS and stained |

| | |
|---|---|
| | with Zombie NIR Viability dye (Biolegend). Cells were then washed and resuspended in staining buffer (HBSS with 2 mM EDTA, 0.5% BSA, and 5% rat serum) with a saturating concentration of anti-CD16/32 (93). Macrophage layers were gently washed five times with warm PBS (with Mg2+ and Ca2+) to remove non cell-associated S. pyogenes and then cells were detached with Accutase (Biolegend). Cells were resuspended in cold PBS with 2 mM EDTA and transferred to low-bind 96-well plates for staining with Zombie NIR Fixable Viability Dye (Biolegend). |
| Instrument | BD FACSCalibur<br>Beckman Coulter Cytoflex<br>Thermo Fisher Attune NxT flow cytometer |
| Software | CytExpert – Version 2.4<br>BD CellQuest Pro – Version 5.1<br>Attune NxT Software – Version 4.2<br>FlowJo – Version 10.6.2 |
| Cell population abundance | No cell sorting was performed |
| Gating strategy | Neutrophils were gated to determine live singlets and defined as CD45+, CD11b+ and Ly6G+. Unstained S. pyogenes controls were used for gating, and a final concentration of 0.2% Trypan Blue or 25 µg/ml Ethidium Bromide, added two minutes before acquisition, was used to determine intracellular bacteria by quenching and/or Förster resonance energy transfer (FRET) of fluorescence from extracellular bacteria. |

☒ Tick this box to confirm that a figure exemplifying the gating strategy is provided in the Supplementary Information.
